# Managing electron-counted MicroED data

**DOI:** 10.1063/4.0000960

**Published:** 2025-10-27

**Authors:** Johan Hattne, Michael W Martynowycz, Max T B Clabbers, William Nicolas, Tamir Gonen

**Affiliations:** 1 Howard Hughes Medical Institute, Los Angeles, CA, USA; 2 University of California, Los Angeles, CA, USA

## Abstract

In microcrystal electron diffraction (MicroED) a series of diffraction images is recorded from a continuously rotating crystal in a transmission electron microscope. Recent electron-counting direct detectors have significantly improved data accuracy while reducing acquisition time. These cameras capture diffraction images at higher frame rates, increasing the demands on data management. They often rely on specialized image formats, which require processing tools to be adapted. Ensuring that metadata remains complete and accurate throughout data collection and processing is essential not only for current analysis but also for future reprocessing with improved methods. This poses a challenge in MicroED, as most microscopes and detectors are not specifically designed for diffraction data collection and metadata often needs to be supplemented by the user. With increasing data volumes, computational efficiency and robust data management are becoming critical to avoid bottlenecks in structure determination. Here, we outline how past experience has shaped our approach to automating metadata bookkeeping for electron- counted data, setting the stage for future improvements that will further streamline the path from MicroED data to structure.

